# Growth assessment in diagnosis of Fetal Growth Restriction. Review

**Published:** 2014-06-25

**Authors:** AR Albu, IA Horhoianu, MC Dumitrascu, V Horhoianu

**Affiliations:** „Carol Davila" University of Medicine and Pharmacy; Bucharest University Emergency Hospital, Department of Obstetrics and Gynecology

**Keywords:** fetal growth restriction, growth charts, percentiles, ultrasound, growth potential

## Abstract

Abstract

The assessment of fetal growth represents a fundamental step towards the identification of the true growth restricted fetus that is associated to important perinatal morbidity and mortality. The possible ways of detecting abnormal fetal growth are taken into consideration in this review and their strong and weak points are discussed. An important debate still remains about how to discriminate between the physiologically small fetus that does not require special surveillance and the truly growth restricted fetus who is predisposed to perinatal complications, even if its parameters are above the cut-off limits established. In this article, we present the clinical tools of fetal growth assessment: Symphyseal-Fundal Height (SFH) measurement, the fetal ultrasound parameters widely taken into consideration when discussing fetal growth: Abdominal Circumference (AC) and Estimated Fetal Weight (EFW); several types of growth charts and their characteristics: populational growth charts, standard growth charts, individualized growth charts, customized growth charts and growth trajectories.

Abbreviations:

FGR = Fetal growth restriction; IUGR = Intrauterine Growth Restriction; SGA = small for gestational age fetus; EFW = estimated fetal weight; AC = abdominal circumference; SD = Standard Deviation; SFH = Symphyseal-fundal height; US = ultrasound; 2D = bidimensional; 3D = tridimensional; RCOG = Royal College of Obstetricians and Gynecologists; FL = femur length; BPD = biparietal diameter; BW = birth weight; IGA = Individualized Growth Assessment; PIH = Pregnancy Induced hypertension; PE = Preeclampsia; NICU = Neonatal Intensive Care Unit.

## Introduction

Fetal growth restriction (FGR) or intrauterine growth restriction (IUGR) is defined today as the failure of the fetus to achieve its growth potential and represents one of the most debated issues in obstetrics, being in continuous search for improvement in terms of definition, classification, diagnosis and management [**1**]. 

 The first step in identifying IUGR is the assessment of fetal growth by comparing the fetal parameters at a certain moment of pregnancy to the expected fetal parameters according to various referentials for the respective gestational age. The main directions towards assessing fetal growth state can be clinical (e.g. the assessment of symphyseal-fundal height) and paraclinical (e.g. ultrasound biometry). A cut-off for a certain parameter is established and values below are signalling an impaired growth. 

 The next step after the determination of ineffective growth is to distinguish between a physiologically small fetus, small for gestational age fetus (SGA) and FGR – which is pathological – with the use of additional tools like Doppler ultrasound sampling of fetal arterial or venous compartments, amniotic fluid volume assessment and other biophysical methods (like computerized CTG) with the goal of indicating the actual state of the small fetus. 

 In order to assess the growth of a fetus we need a parameter – ultrasonic parameters being widely used, a referential – e.g. a growth chart with the expected interval values of a certain parameter for a given gestational age and a cut-off value.

 The ultrasound limit for SGA or FGR is arbitrarily fixed at an estimated fetal weight (EFW) or abdominal circumference (AC) under the 3rd, 5th, 10th percentile or below -2 Standard Deviation (SD) from the population standard or reference [**2**]. When considering the 3rd percentile as a cut-off, fetuses below have a higher risk to be growth-restricted but a great number of GR fetuses are omitted. This could lead to an increased number of pregnancies omitted from the definition and therefore inconsistently managed and associated to bad outcome. Clausson et al. showed that fetuses found between the 3rd and the 10th percentile, the so-called "mildly growth-restricted" are at increased risk for complications [**3**]. Furthermore, it might be useful to consider as growth restricted, fetuses whose growth trajectories significantly decrease from an ultrasound examination to another, switching from a superior percentile to an inferior one even if the estimated weight or abdominal circumference remains above the 10th percentile [**1**]. 

 In summary, the definition of FGR seems to be more a dynamic one and the diagnosis should be made after serial evaluations of fetal growth as well as of fetal state of health parameters [**4**]. For the very precocious FGR, especially if there is a symmetrically affected fetus, the genetic factor or the intrauterine infections can be incriminated. In the asymmetric FGR, where placental insufficiency accounts for most of the cases, we still need reliable predictive tests to identify pregnancies at risk to develop the pathology and the surveillance of growth in the late second trimester and, the third trimester continues to be the mainstay in the detection of the condition. The purpose of this study is to review current concepts in the growth assessment as part of the diagnosis of FGR.

 Clinical methods of fetal growth estimation: Symphyseal – Fundal Height and Leopold Maneuver

 Symphyseal-fundal height (SFH) measurement was historically used in the assessment of gestational age but with the advent of ultrasound dating of pregnancy, it lost its interest. However, in places where ultrasound (US) tools are not available in every obstetrical service, it still remains an important tool for pregnancy week assessment. In a dated pregnancy, a modified SFH can be the first parameter to raise the question of FGR, some guidelines recommend it as a screening tool from the second or the third trimester and a significant proportion of cases is followed by the confirmation through ultrasound scan [**5**]. 

 Starting from the 1980s, authors confirmed the usefulness of SFH in detecting SGA [**6**]. As reviewed by McDermott et al., in the general population, SFH is able to provide a good description of fetal growth, with a detection rate of FGR between 17% and 93% and a sensitivity of 65%, for a 50% false positive rate, with variations depending on the methodology used and the presence of factors like high maternal BMI, uterine tumors (leyomioma) or multiparity [**7-8**]. Unsuitability of SFH measurment is apliable to high risk pregnancies, like those identified from the first trimester through history or presence of predictive markers that lead to serial US scan starting with week 26-28 [**9**].

 In 1999, Gardosi and Francis showed that plotted SFH on customized charts nearly doubled the detection rate of growth abnormalities [**10**]. In 2009, Morse et al. presented a standardized protocol of SFH measurement by using non-elastic tape and plotting the values on customized charts starting with week 26-28. The key conclusions of the study are that a single SFH detection below the 10th percentile or a static or slow growth curve represents indication to ultrasound assessment of growth together with Doppler analysis performed by the materno-fetal specialist [**5**]. Furthermore, in 2012, Roex et al. considered that this method is doubling the antenatal detection of SGA fetuses in nulliparous women [**11**]. Yet, the 2012 Cochrane analysys by Peter et al. showed no benefit of using SFH for the detection of FGR, none the less fundal height measurements plotted on customised growth charts are still recommended by the RCOG Green Top Guidelines [**9,12**]. 

 Ultrasound methods of estimating the fetal growth

 Growth ultrasonic parameters 

 The accurate assessment of fetal weight is an essential component in prenatal and perinatal care. There is no consensus in using a universal method of measure because of the heterogeneous population and the multitude of physiological and pathological factors influencing the growth of the fetuses.

 Ultrasound exams are reliable diagnostic tools and fetal biometry is the gold standard in detecting abnormal fetal growth. Starting from the 80s various ultrasound models of assessing fetal growth have been proposed. Deter et al. introduced the 3D parameters but soon 2D biometry became the prefered investigative tool due to the fact that it is easier to perform compared to 3D US [**13**]. Various researcher groups tried to establish a reliable formula to accurately estimate the real fetus weight and the calculation method was verified through evaluations performed few days before birth [**14**]. In the United States, Hadlock et al. introduced three formulas based upon biometric parameters like abdominal circumference (AC), femur length (FL), biparietal diameter (BPD) [**15**]. Over the years, further estimation formulas for fetal weight have been introduced: Campbell, Kehl, Siemer, Robson [**14**]. Among them, the sensitivity for the detection of SGA ranged from 72% to 100%, and the specificity was from 41% to 88%. Hadlock C formula for birth weight (BW) estimation had the best sensitivity/specificity trade-off for the detection of SGA ((log10 BW = 1.335 − 0.0034x(AC)x(FL) + 0.0316x(BPD) + 0.0457(AC) + 0.1623(FL) )) [**14**].

 The 2D estimation of fetal weight is routinely performed and in order to identify the impaired growth we must rely upon fetal growth charts for different parameters – AC, EFW on which the ultrasound estimates can be recorded.

 A. Growth charts – key concepts

 Some basic concepts must be considered before performing an evaluation of the fetal growth. As discussed earlier, we cannot define a parameter as being small without comparing it to a referential. Growth references are descriptive indicators, which represent the outcomes of all the pregnancies, both normal and pathologic. On the other side, growth standards are normative indicators where only the outcomes of normal pregnancies are considered. These notions gain an even more important value when we try to reveal the presence of real pathological growth and in the construction of a referential that should categorize a fetus as normal or growth restricted [**16**]. Furthermore, we can use neonatal referentials that are based on anthropometric parameters registered after birth for a given gestational age (e.g. birth weight) or fetal referentials that are based on ultrasound estimation of fetal weight and biometrical parameters [**16**]. 

 It has been shown that prematurity (birth before 37 weeks of gestation), whether spontaneous or iatrogenic, is often associated with smaller weights and that fetuses who continue to develop in utero have greater weights than those prematurely born at the same gestational age [**17**]. Fetal growth restriction is frequently linked to preterm birth and, as a result, at a lower gestational age, the growth curves based on birth weight are flattened and may reduce the number of SGA cases detected [**18,19**]. These populational birth weight growth charts are based on large cohorts of living births (both normal and pathologic) at a certain gestational age and some are only adjusted for baby gender [**17,20**]. In complicated pregnancies (fetal anomalies, hypertension, diabetes) the growth is impaired and in order to achieve a correct diagnosis we should compare the estimated fetal weight to only normal models of growth. Therefore, the idea of reporting the actual weight to ultrasound estimated weight charts in normal outcome pregnancies became prevalent in order diagnose cases of growth restriction. We concluded that the best referential for the assessment of the growth of a fetus are intrauterine estimated ultrasound growth standards. 

 Population growth standards are based on the analysis of large samples of fetuses starting from the principle that all the fetuses have the same growth potential. Yet, the physiological and environmental factors that influence the fetus’ growth are not being adjusted for, so, more than the population standards, the customized standards which have been adjusted for maternal and fetal characteristics and are supposed, came to refine the sensibility of the detection of true growth-restricted fetuses [**21**].

 Individualized growth charts

 a. The fetus as its own control

 In 1984, Rossavik and Deter launched a mathematical model of fetal growth introducing two new parameters, head cube and abdominal cube. The new growth curve model was able to predict changes in the values of the chosen parameter with the advancement of the pregnancy [P=c(t)k+s(t) where P is the growth of the biometric parameter chosen, t = time of pregnancy when the observation was made and c, k, s are the model coefficients] [**22**]. Over the next years, the two authors published models of growth for multiple fetal parameters: head cube, abdominal cube, head and abdomen profile area, femoral diaphisis, etc. Individual measurements from fetuses <26 weeks of gestation, taken from two ultrasound scans separated by a 4-8 weeks interval, could be able to predict the respective third trimester growth patterns with low errors. These results were confirmed in 1989 by Simon et al. and in 2004 by Deter et al., the latter introducing the concept of evaluation of growth by using "each fetus as its own control" and further defining the term Individualised Growth Assessment (IGA) [**23,24**].

 Although appealing, the method found its contestants as it assumed a perfect fetal growth during the first and second trimester of pregnancy, whereas pathologic and environmental factors are influencing growth as early as the first trimester [25,26]. The two ultrasound performed <26 weeks used as referential could artificially flatten the growth trajectory leading to a decreased number of growth restricted fetuses identified [**27**]. Measuring errors could also be present and could alter the prediction of the term weight [**27**]. Furthermore, the comparison between the conventional growth curves and the mathematical models showed that the former do not require complex calculations and are conceptually simpler and easier to use [**28**].

 b. Customized growth charts

 The debate around "fetal growth potential" assessment started with the studies on customized growth charts performed by Gardosi et al. and their benefits and weak points continue to be discussed [**21**]. The pregnancy characteristics are used in order to calculate the Term Optimal Weight - the weight that the baby is predicted to achieve in the absence of pathological influences at 40 weeks of pregnancy [**33**].

 The fetal growth standard should be "customized" for each fetus, including physiological characteristics of mother and fetus like race, ethnicity, maternal height and weight at booking, parity and sex of the fetus [**21,27**]. These growth curves must be based upon intrauterine fetal growth, as premature birth will result in a fetus with a lower weight than the fetus that continues to grow in utero [**29,30**]. It seems that in the pathological pregnancies, the in utero growth is affected and premature birth represents a way of escaping the unfavorable milieu that would lead to further fetal distress [**31**]. Another important issue in defining the fetal growth potential is the exclusion of pathology – like hypertension or diabetes and smoking, which would affect the birthweight [**32**]. With the aid of customized charts we can identify a group of truly growth restricted fetuses that have not been recognized through population charts and present a higher perinatal risk. Furthermore, another group of previously small fetuses would be considered normal and having a good outcome. 

 In the beginning of the 2000s, Gardosi et al. developed a free downloadable software, "the GROW Software", that takes into account several physiological variables to assess intrauterine growth charts [**33**]. Taking into account populational differences, various fetal growth potential were developed in countries like United Kingdom, Australia, New Zeeland, France, United States, Spain [**27,34-38**]. Although promising, the customized growth charts were contested by some researchers [**39,40**]. However, their apparent benefits are more likely to have been derived from their incorporation of intrauterine-based (EFW) reference values at preterm ages than their adjustment for maternal characteristics. 

 c. Growth trajectories – new promising tools

 The value of detecting true small babies is real but what is questionable is the prediction of customized growth curves for perinatal morbidity and mortality, because a great amount of fetuses are premature and the prematurity complications superpose over the IUGR complications. 

 However, the usefulness of fetal growth trajectories is real in detecting the anomalies of growth patterns, which are important in detecting growth-impaired fetuses whose echografic parameters are still above the 10th percentile but decrease from an ultrasound exam to another.

 In a recent study [**41**], two fetal growth trajectories (normal and pathologic) were described in a group of fetuses with impaired growth, helping in the detection of undergrown fetuses at real risk of poor neonatal outcome. Although it has some limitations (it includes a cohort of fetuses already diagnosed with abnormal growth), the strong points of the study were the dynamic assessment of growth and the individual approach based on repeated examination of the same fetus.

## Conclusions

The diagnosis of FGR has as a first step the identification of impaired fetal growth and the confirmation is made with tools like fetal arterial and venous Doppler assessment, biophysical indices. The failure of FGR identification is an important cause of perinatal morbidity with an increase of the level of adverse perinatal outcome. SFH measurement could be used as a cost-effective method of screening for fetal growth from 20–24 gestation weeks in the appropriate cases. Ultrasound evaluation of fetal growth defines as reliable AC or/and EFW parameters. For the definition of a small fetus, a referential and a cut off value are necessary. Along the years, researchers have tried to find a predictible model of growth for the fetus in order to early detect anomalies of growth. "Fetus as its own control" was a concept of development individualized for each fetus based on the growth pattern from the beginning of pregnancy. The use of customized centiles, seemed to increase the prediction of at risk pregnancies for perinatal mortality and the association with other perinatal complications like PIH, PE, threatened labor, antepartum hemorrhage, emergency cesarian section for fetal distress, low Apgar score and necessity of NICU admission. The latest research patterns purpose of fetal growth – growth trajectories – being promissing in identifying fetuses with an affected development. What is really important is to distinguish between small fetuses with poor periantal outcome and physiologically small fetuses and perform a protocol of increased surveillance on the risk fetuses.
